# Latitudinal Patterns in European Seagrass Carbon Reserves: Influence of Seasonal Fluctuations versus Short-Term Stress and Disturbance Events

**DOI:** 10.3389/fpls.2018.00088

**Published:** 2018-02-01

**Authors:** Laura M. Soissons, Eeke P. Haanstra, Marieke M. van Katwijk, Ragnhild Asmus, Isabelle Auby, Laurent Barillé, Fernando G. Brun, Patricia G. Cardoso, Nicolas Desroy, Jerome Fournier, Florian Ganthy, Joxe-Mikel Garmendia, Laurent Godet, Tiago F. Grilo, Petra Kadel, Barbara Ondiviela, Gloria Peralta, Araceli Puente, Maria Recio, Loic Rigouin, Mireia Valle, Peter M. J. Herman, Tjeerd J. Bouma

**Affiliations:** ^1^Department of Estuarine and Delta Systems, NIOZ Royal Netherlands Institute for Sea Research, Utrecht University, Yerseke, Netherlands; ^2^Department of Environmental Science, Institute for Water and Wetland Research, Radboud University, Nijmegen, Netherlands; ^3^Alfred-Wegener-Institut, Helmholtz-Zentrum für Polar- und Meeresforschung, Bremerhaven, Germany; ^4^Institut Français de Recherche pour l’Exploitation de la Mer – Laboratoire Environnement-Ressources d’Arcachon, Arcachon, France; ^5^Equipe Mer-Molécules-Sante EA 2160, Faculté des Sciences et des Techniques, Université de Nantes, Nantes, France; ^6^Departamento de Biología, Área de Ecología, Facultad de Ciencias del Mar y Ambientales, Universidad de Cádiz, Cádiz, Spain; ^7^Department of Life Sciences, Marine and Environmental Research Centre, University of Coimbra, Coimbra, Portugal; ^8^Institut Français de Recherche pour l’Exploitation de la Mer – Laboratoire Environnement et Ressources, Dinard, France; ^9^Centre National de la Recherche Scientifique, UMR 7208 Biologie des Organismes et Ecosystèmes Aquatiques, Paris, France; ^10^Centro Tecnológico Experto en Innovación Marina y Alimentaria-Tecnalia, Marine Research Division, Pasaia, Spain; ^11^Centre National de la Recherche Scientifique, UMR 6554 Littoral, Environnement, Teledetection, Geomatique-Nantes Géolittomer, Université de Nantes, Nantes, France; ^12^Marine and Environmental Sciences Centre, Laboratorio Maritimo da Guia, Faculdade de Ciências da Universidade de Lisboa, Cascais, Portugal; ^13^Environmental Hydraulics Institute “IH Cantabria”, Universidad de Cantabria, Santander, Spain; ^14^Escuela de Gestión Ambiental, Pontificia Universidad Católica del Ecuador Sede Esmeraldas – PUCESE, Esmeraldas, Ecuador

**Keywords:** carbon reserves, European Atlantic coast, latitude, resilience, *Zostera noltei*, climate setting, stress events

## Abstract

Seagrass meadows form highly productive and valuable ecosystems in the marine environment. Throughout the year, seagrass meadows are exposed to abiotic and biotic variations linked to (i) seasonal fluctuations, (ii) short-term stress events such as, e.g., local nutrient enrichment, and (iii) small-scale disturbances such as, e.g., biomass removal by grazing. We hypothesized that short-term stress events and small-scale disturbances may affect seagrass chance for survival in temperate latitudes. To test this hypothesis we focused on seagrass carbon reserves in the form of starch stored seasonally in rhizomes, as these have been defined as a good indicator for winter survival. Twelve *Zostera noltei* meadows were monitored along a latitudinal gradient in Western Europe to firstly assess the seasonal change of their rhizomal starch content. Secondly, we tested the effects of nutrient enrichment and/or biomass removal on the corresponding starch content by using a short-term manipulative field experiment at a single latitude in the Netherlands. At the end of the growing season, we observed a weak but significant linear increase of starch content along the latitudinal gradient from south to north. This agrees with the contention that such reserves are essential for regrowth after winter, which is more severe in the north. In addition, we also observed a weak but significant positive relationship between starch content at the beginning of the growing season and past winter temperatures. This implies a lower regrowth potential after severe winters, due to diminished starch content at the beginning of the growing season. Short-term stress and disturbances may intensify these patterns, because our manipulative experiments show that when nutrient enrichment and biomass loss co-occurred at the end of the growing season, *Z. noltei* starch content declined. In temperate zones, the capacity of seagrasses to accumulate carbon reserves is expected to determine carbon-based regrowth after winter. Therefore, processes affecting those reserves might affect seagrass resilience. With increasing human pressure on coastal systems, short- and small-scale stress events are expected to become more frequent, threatening the resilience of seagrass ecosystems, particularly at higher latitudes, where populations tend to have an annual cycle highly dependent on their storage capacity.

## Introduction

Seagrasses are flowering plants, adapted to the marine environment (Les et al., 1997), forming extensive and highly productive meadows worldwide (Short et al., 2007). Throughout the year, temperate seagrass meadows are submitted to various abiotic and biotic variations: (i) seasonal fluctuations related to variations in light and temperature controlling their presence and seasonal growth (Dennison, 1987; Duarte, 1991; Olesen and Sand-Jensen, 1993; Ochieng et al., 2010; Marbà et al., 2012); (ii) short-term stress events such as local nutrient enrichment leading to eutrophication; and (iii) small-scale disturbances such as biomass removal by grazing (e.g., by birds or sea turtles), jointly affecting their resilience and survival (Burkholder et al., 2007; Macreadie et al., 2014).

The capacity of seagrasses to respond and recover from stresses and disturbances (Charpentier et al., 2005; Godet et al., 2008) depends on their clonal growth strategy (i.e., potential rhizome elongation rate; Macreadie et al., 2014), their seasonal growth (Sordo et al., 2011; Soissons et al., 2016), their seed production (Van Tussenbroek et al., 2016), their high productivity (Plus et al., 2005; Ribaudo et al., 2016), and their ecosystem engineering capacity (McGlathery et al., 2012). Seagrasses can acclimate their morphological, physiological, and mechanical traits to local conditions (Peralta et al., 2005, 2006; Cabaço et al., 2009; de los Santos et al., 2010, 2013; La Nafie et al., 2013; Soissons et al., 2018). This plasticity improves their resilience under threats. However, in the current context of climate change and concomitant increasing anthropogenic pressure on coastal ecosystems, it can be assumed that environmental stressors are going to increase, on types and/or intensity. Stressor combinations can generate synergistic, additive, or antagonistic effects on seagrass populations, deteriorating environmental conditions for the resistance and resilience of seagrass ecosystems. The decline of seagrass ecosystems has been described worldwide (Orth et al., 2006; Waycott et al., 2009). If changes in environmental conditions result in a decrease of seagrass resilience, their very valuable ecosystem services may not only decrease but also disappear (Scheffer et al., 2001, 2009; Carr et al., 2012).

Seagrasses overcome periods of low-light availability like the winter months or short-term light deprivation events (Burke et al., 1996; Govers et al., 2015) using their carbon reserve, in the form of non-structural carbohydrates (i.e., starch and/or soluble sugars) gained during their growing season (Madsen, 1991; Alcoverro et al., 1999; Lee et al., 2007; Olivé et al., 2007). Non-structural carbohydrates are usually stored when photosynthesis exceeds the carbon demand for growth and respiration (Madsen, 1991). The magnitude of the carbon reserve needed for seagrass survival depends on abiotic factors such as, i.e., temperature and light availability, but also on internal factors affecting the carbon balance, such as respiration and growth (Madsen, 1991; Alcoverro et al., 2001; Govers et al., 2015). Survival after winter thus depends on the plants’ capacity to build up their carbon reserves, particularly in the form of starch, during their growing season (Govers et al., 2015).

Studies have shown that seagrass carbon balance, and therefore conditions to favor storage of non-structural carbohydrates depend on light irradiance, daylight length (photoperiod), temperature, weather, and hydrodynamic conditions (Marsh et al., 1986; Burke et al., 1996; Alcoverro et al., 2001; Olivé et al., 2007; Govers et al., 2015). To our knowledge, previous studies did not include a spatial dimension, such as latitudinal patterns, to understand carbon storage in seagrasses. Latitudinal gradients affect variables like day length, temperature, and winter intensity, which are important in defining the carbon balance of seagrasses (Alcoverro et al., 2001). Hence, the capacity of a seagrass species to store non-structural carbohydrates such as starch and constitute a carbon reserve may be influenced by its distribution along latitudinal gradients.

Short-term stress events and small-scale disturbances have also very high potential in affecting seagrass carbon reserves, particularly when occurring during their growing season. Short-term stress events may be linked to high turbidity pulses or excess nutrient enrichment [leading to nitrate or ammonium toxicity (van Katwijk et al., 1997; Brun et al., 2002)]. Small-scale disturbances may reduce seagrass abundance and biomass, by, e.g., grazing, trampling, or digging. Such short-term stress and disturbance events may occur separately or act simultaneously, increasing the risk of carbon reserve depletion, in severe cases, or modifying the plant’s capacity to store non-structural carbohydrates such as starch. However, these effects are not only expected to depend on the magnitude and type of events, but also on the timing of occurrence. It could thus be hypothesized that the consequences of short-term stress or disturbance events may be different if they would happen at the beginning or at the end of the seagrass growing season.

To clarify these questions, (i) we used the dwarf eelgrass *Zostera noltei* Hornem. as a model species to test how its carbon reserves (i.e., starch content) might differ depending on its distribution along a latitudinal gradient at key stages of the growing cycle (i.e., at the beginning, the peak, and the end of the growing season). With this, we aimed to evaluate the influence of seasonal fluctuations (i.e., air/water temperatures during the growing season and from the previous winter period, daylight length) on *Z. noltei* starch content. Additionally, (ii) we assessed, through a manipulative experiment on two *Z. noltei* meadows located at a single latitude in the Netherlands, the effect of short-term stress event (i.e., local nutrient enrichment), small-scale disturbance (i.e., above-ground removal), and their combination on their capacity to store carbon in the form of starch along their growing season. From this, (iii) we discussed how short-term stress and disturbance events might affect the plant’s seasonal carbon reserve (i.e., starch content) and thus their capacity to withstand seasonal fluctuations, as well as their long-term resilience and survival depending on their distribution along a latitudinal gradient.

## Materials and Methods

### Study Sites

**FIGURE 1:**
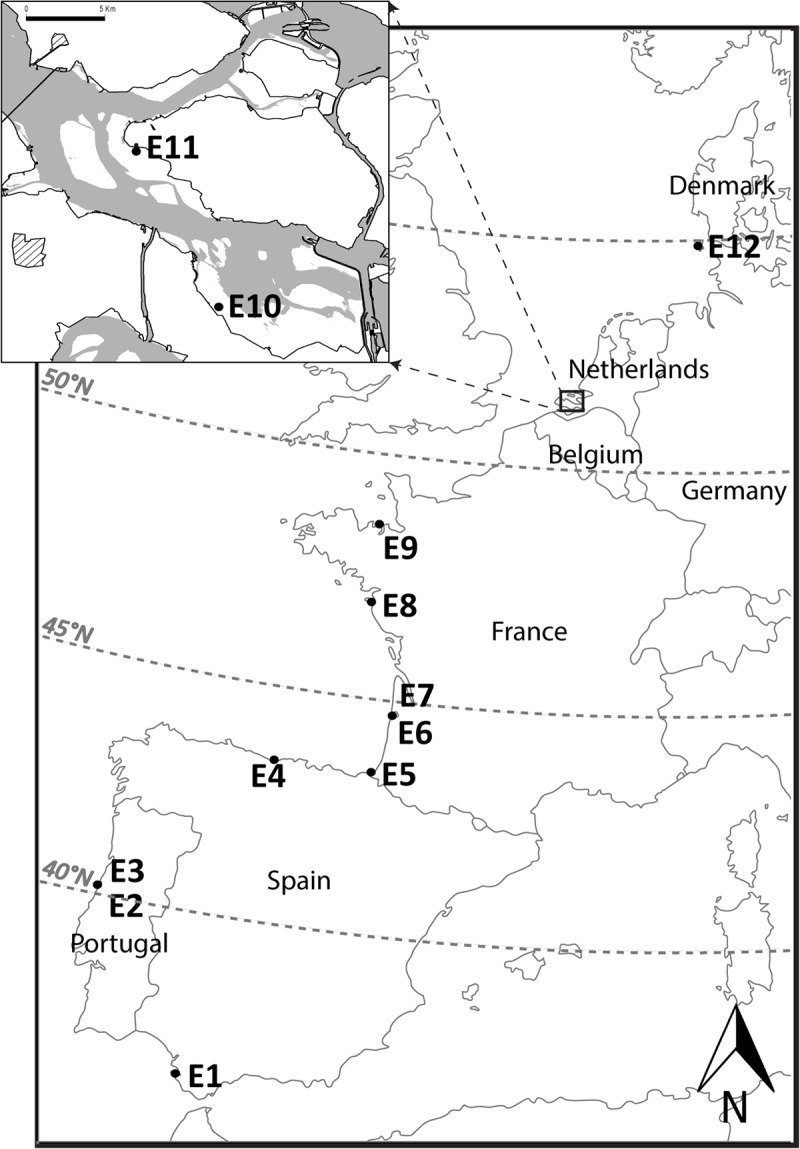
Localization of the sampling sites along the Western European coast, from south to north: E1 Cadiz (Spain); E2 and E3 Mondego estuary (Portugal); E4 Santander (Spain); E5 Bidasoa esturary (France); E6 and E7 Arcachon Bay (France); E8 Noirmoutier (France); E9 St-Jacut-de-la-mer (France); E10 and E11 The Oosterschelde (Netherlands); and E12 Sylt (Germany); Zoom in the case study sites (E10 and E11).

In order to cover a latitudinal gradient, seagrasses were sampled at 12 sites along the European coastline, at three different times over the growing season: at the beginning, at the peak, and at the end of the growing season (**Table 1**). Sites were selected following a latitudinal gradient from south (warmer) to north (colder), being: E1 Cadiz (Spain); E2 and E3 Mondego estuary (Portugal); E4 Santander (Spain); E5 Bidasoa estuary (France); E6 and E7 Arcachon Bay (France); E8 Noirmoutier (France); E9 St-Jacut-de-la-mer (France); E10 and E11 The Oosterschelde (Netherlands); and E12 Sylt (Germany) (**Figure 1** and **Table 1**). The samples were always on healthy and well-developed *Zostera noltei* meadow found in intertidal areas (as determined by local experts). Sampling dates were not identical between locations, as the beginning and duration of the growing season are dependent on local conditions such as temperature and light availability, which are latitude-dependent. To get a comparable set of data for the different stages in the growing season (i.e., beginning, peak, and end), the exact sampling dates were determined by local experts (**Table 1**).

**Table 1:** Site characteristics and sampling dates.

	Site number and names	Coordinates	Start–end experimental dates			Temperature (°C)				Daylight length (hours in decimals)				Ever green Y/N
			Beginning	Peak	End	Past winter	Beginning	Peak	End	Past winter	Beginning	Peak	End	
E1	Cadiz Bay, Spain	N 36 30^′^ W 6 10^′^	2 July	9 September	6 November	13.45	–	26.1 ± 0.08	20.6 ± 0.08	9.71	14.67	12.67	10.5	Y
E2	Upstream – Mondego estuary, Portugal	N 40 8^′^ W 8 50^′^	28 May	13 August	10 November	10.0	17.6 ± 0.1	20.8 ± 0.09	18.8 ± 0.07	10.03	14.67	13.83	10.17	Y
E3	Downstream – Mondego estuary, Portugal	N 40 8^′^ W 8 50^′^	28 May	13 August	10 November	10.0	17.3 ± 0.1	20.6 ± 0.1	18.7 ± 0.08	10.03	14.67	13.83	10.17	Y
E4	Santander, Spain	N 43 25^′^ W 003 48^′^	13 June	11 August	8 October	11.5	17.2 ± 0.06	22.6 ± 0.03	19.9 ± 0.03	9.03	13.83	14.2	11.47	Y
E5	Bidasoa estuary, France	N 43 21^′^ W 001 46^′^	29 May	7 August	11 October	9.7	–	23.3 ± 0.1	20.6 ± 0.1	9.02	15.08	14.37	10.5	Y
E6	Germanan – Arcachon Bay, France	N 44 42^′^ W 001 8^′^	27 May	13 August	27 October	8.7	17.1 ± 0.09	23.2 ± 0.07	18.9 ± 0.08	8.87	15.17	14.21	10.5	Y
E7	Hautebelle – Arcachon Bay, France	N 44 43^′^ W 001 09^′^	27 May	13 August	27 October	8.7	17.1 ± 0.09	23.2 ± 0.07	19.0 ± 0.08	8.87	15.17	14.21	10.5	Y
E8	Noirmoutier, France	N 46 98^′^ W 002 21^′^	27 June	25 July	11 September	9.5	20.4 ± 0.09	21.3 ± 0.09	21.1 ± 0.07	8.55	15.92	15.25	12.88	N
E9	St-Jacut-de-la-mer, France	N 48 36^′^ W 002 11^′^	25 June	29 July	20 August	7.8	17.3 ± 0.1	20.4 ± 0.1	20.1 ± 0.2	8.35	16.15	15.25	14.18	N
E10	Oostdijk – Oosterschelde, Netherlands	N 51 26^′^ E 004 05^′^	11 June	19 August	26 September	6.8	19.2 ± 0.1	20.3 ± 0.1	19.0 ± 0.1	7.9	16.53	14.5	12.03	N
E11	Dortsman – Oosterschelde, Netherlands	N 51 34^′^ E 003 59^′^	12 June	20 August	25 September	6.8	18.0 ± 0.1	20.2 ± 0.08	18.3 ± 0.07	7.9	16.53	14.5	12.03	N
E12	Sylt, Germany	N 54 54^′^ E 008 19^′^	–	30 July	12 September	3.3	–	22.2 ± 0.1	17.9 ± 0.09	7.4	–	16.05	12.98	N

*The past winter temperatures and daylight length (in hours) are averaged over the winter months 2013–2014. The water temperatures are averaged (±SE) from measurements taken by the HOBO-pendant loggers over 1 month around the sampling dates at all sites (data collected in 2014)*.

### Experimental and Sampling Design

#### The Influence of Seasonal Fluctuations and Latitude on Carbon Reserves

For each sampling date and at each site (**Table 1**), seagrass samples (*n* = 5) were collected by using 10 cm diameter PVC cores inserted into the sediment. The seagrass samples were briefly washed *in situ* and stored in wet tissues for preservation during transportation to the NIOZ (Royal Netherlands Institute for Sea Research) in Yerseke, Netherlands. In the laboratory, the samples were carefully washed a second time with freshwater to remove all remaining sediment, algae, and epiphytes (scraped with a razor blade). Then, on every sample, rhizomes were carefully separated from the roots and leaves, and subsequently freeze-dried for carbon reserves (i.e., starch content) analyses (rhizomes only).

Water temperature was continuously monitored over the study period at every sites. Water temperature was measured using two HOBO Pendant Temperature loggers (64k – UA-002-64, ONSET) at a frequency of 1 measurement every 30 min. For every study site, two loggers were placed within the study area. Air temperature and daylight length in hours from the past winter months and during the experimental period were obtained from local weather stations and available climate and weather databases online (i.e., www.aemet.es for Spain; www.ipma.pt for Portugal; www.meteofrance.fr for France, www.buienradar.nl for Netherlands; www.dwd.de for Germany; and www.timeanddate.com for all). For each site, past winter temperature and daylight length were averaged from December 2013 until February 2014. Daylight length (hours expressed in decimals) was averaged over 1 month around the sampling date (**Table 1**).

#### The Influence of Short-Term Nutrient Enrichment and Small-Scale Disturbances on Carbon Reserves

Seagrass carbon reserves’ response to short-term stress (nutrient enrichment) and small-scale disturbance (above-ground biomass removal) was evaluated only at two sites located at the same latitude (Oosterschelde, Netherlands; Sites E10 and E11; **Figure 1** and **Table 1**). Sites E10 and E11 both display similar average exposure characteristics (Suykerbuyk et al., 2016) but experience different wind orientation and condition making them visibly different during the experimental period. At E10, the sediment particle median diameter (D50) was 78.1 ± 43.5 μm with 43.5 ± 0.6% of fine particles (<63 μm); whereas at E11 the D50 was 94.7 ± 13.8 μm with 22.9 ± 1.4% of fine particles (<63 μm) and the presence of marked sand ripples (personal observation). The study was designed as a factorial experiment with four treatments (*n* = 5 for each treatment): Control, i.e., undisturbed-no nutrient (C); undisturbed with nutrient (CN); disturbed-no nutrient (D); disturbed with nutrient (DN). The same experimental design was implemented at both sites three times over the growing season (at the beginning: 14 May–12 June; the peak: 22 July–20 August and the end: 27 August–26 September 2014).

##### Plot installation

To minimize edge effects, the experimental plots at both sites were randomly allocated in the middle of the seagrass meadows, providing a minimum distance of at least 5 m between plots to avoid an experimental nutrient release overlap. Every experimental plot was delimited by two bamboo sticks placed 1 m apart and included an inner circle of 30 cm diameter for the treatments, delimited by 10 metal sticks. To avoid contamination from previous experimental setups, the experimental areas, at each site, were different and independent for each of the three experimental periods.

##### Short-term nutrient enrichment

Nutrient enrichment was simulated by placing small bags of slow release fertilizers (Osmocote, N:P:K = 15:9:12) in the upper layer of sediment around the (CN) and (DN) 30 cm diameter inner circles by using the metal sticks as anchors. The fertilizer bags were made of panty hoses, containing 10 g of slow release fertilizer each. Experimental plots with nutrient treatments [(CN): *n* = 5 and (DN): *n* = 5 plots per experiment] had a total of 10 bags per plot, receiving in total 100 g of slow-release fertilizer per plot (i.e., 1.4 kg m^-2^ slow release fertilizer), which corresponds to a high and potentially toxic enrichment (Govers et al., 2014).

##### Small-scale disturbances

Disturbances of above-ground biomass were created by clipping the leaves, leaving the below-ground and sheaths in place inside the (D) and (DN) (*n* = 10) 30 cm diameter inner circles. This type of disturbance was chosen to mimic the effect of over-grazing creating gaps in seagrass meadows and allowing direct regrowth measurements within the 4 weeks long experiments. All seagrass material removed at gap creation was kept in individual bags for biomass measurements. In order to minimize seagrass regrowth based on carbon reserves outside the experimental 30 cm inner circles (i.e., lateral carbon transfer), rhizomes around the inner circles were initially cut around the 30 cm inner circles (including control plots).

##### Seagrass samples

At the start of each experiment (at sites E10 and E11 only), initial seagrass samples (*n* = 5) were collected close to the experimental plots by using 10 cm diameter PVC cores inserted into the sediment. At the end of each experimental period, all plots were harvested by using the same 10 cm diameter PVC cores inserted into the sediment randomly within the inner 30 cm diameter inner circle. Seagrass samples were washed and processed in the same way as for the samples collected along the latitudinal gradient (see description above).

### Measuring Starch Content in Rhizomes for Seagrass Carbon Reserves

Seagrass rhizomes store most of the non-structural carbon reserves in the form of starch (Burke et al., 1996; Zimmerman and Alberte, 1996; Olivé et al., 2007; Govers et al., 2015). Those reserves were evaluated in rhizomes from control samples for all pan-European sites, to investigate the potential of each site to store carbon reserves over their growing season. Carbon reserves were measured in freeze-dried and ground rhizomes of the control samples from the beginning, peak, and end of the growing season of all 12 field sites. In addition, the carbon reserves of the rhizomes samples from all treatments at the Oosterschelde sites (site E10 and E11) were also analyzed to investigate their response to treatments.

To evaluate seagrass carbon reserves, we measured the starch content of seagrass rhizomes. Soluble sugars – glucose, fructose, and sucrose – were extracted out of the plant material by using an 80% ethanol solution. Those ethanol soluble sugars were not used for our analysis. The residue of the extraction was hydrolyzed with diluted hydrochloric acid (3% HCl) to convert starch into glucose. After hydrolysis, the residue was boiled at 100°C for 30 min. Rhizomal starch content was estimated by anthrone assay standardized to D-glucose (Yemm and Willis, 1954) and converted to milligram starch per gram dry plant material. All samples were measured in duplicate and a new calibration curve was prepared for every series of measurements.

### Statistical Analysis

Variations in water temperature and daylight length were tested using a one-way ANOVA followed by Tukey’s *post hoc* tests when significant, considering as variation source the factor “experimental period” (i.e., the growing season stages: beginning, peak, and end). Differences in starch content and above-ground biomass were assessed with a two-way ANOVA using as variation sources the factors “experimental period” and “site”.

For the latitudinal gradient, variations in water temperature and daylight length during the growing season and for the past winter were evaluated with linear regressions, on data split per experimental period. The effect of seasonal fluctuations on starch content was tested by evaluating the existence of linear relationships between past winter temperatures, daylight length, or latitude with starch content at the beginning and the end of the growing season. The linear regressions were separately performed for the two stages of the growing season. For daylight length, an additional linear regression was performed at the peak of the growing season. The relationships between temperature (i.e., water temperature, as measured from the loggers over the experimental period, and winter temperature, collected from weather stations or databases) with latitude were tested with a general linear model.

The effect of treatments (i.e., short-term nutrient enrichment and above-ground removal) on rhizomal starch content for the two treated sites E10 and E11 was tested using a mixed effects model considering “sites” (E10 and E11), “treatment” (C, N, D, ND), and “experimental period” (beginning, peak, and end) as fixed factors and replicates (*n* = 5) as a random factor. Differences between treated plots (N, D, and ND) and control (C) were obtained with *post hoc* Tukey tests. Data normality were tested prior to analysis using the Shapiro–Wilk test. All statistical analyses were realized with R version 3.1.3 (R Core Team, 2013).

## Results

As expected water temperature was highest at the peak of the growing season (ANOVA: *F* = 24.28, *p* < 0.001; Tukey: peak > end > beginning, *p* < 0.001) with a temperature range from 20.2 ± 0.1°C at site E11 up to 26.1 ± 0.1°C at site E1 (**Table 1**). Daylight length significantly decreased at the end of the growing season (ANOVA: *F* = 48.99, *p* < 0.001; Tukey: beginning = peak, *p* = 0.52; beginning > end, *p* < 0.001; peak > end, *p* < 0.001). Daylight length presented a linear decrease from North to South at the beginning and at the end of the season (**Table 2**). Daylight length at the peak of the season and water temperatures along the season (i.e., for all experimental periods) did not show any significant linear relationships with latitude (**Table 2**). However, past winter temperature and daylight length were significantly higher in southern latitudes and lower in northern latitudes (**Tables 1**, **2**).

**Table 2:** Results from the linear regressions between latitude and temperature (water for beginning, peak, and end; air for past winter) or daylight length at the different stages of the growing season (beginning, peak, and end) and for the past winter.

	Temperature				Daylight length			
	*R*^2^	*F*	*p*-value	Equation when sig.	*R*^2^	*F*	*p*-value	Equation when sig.
Beginning	0.20	1.77	0.22		0.7	20.98	0.001^∗^	*y* = 8.46 + 0.15*x*
Peak	0.26	3.58	0.09		0.02	0.14	0.71	
End	0.17	2.10	0.18		0.53	11.48	0.007^∗^	*y* = 3.3 + 0.18*x*
Past winter	0.84	53.75	<0.001^∗^	*y* = 28.7-0.44*x*	0.93	126.11	<0.001^∗^	*y* = 15.6-0.15*x*

*Equations are given only when the linear relationship is significant with: y representing the tested variable (temperature and daylight length) and x representing latitude in all cases. Asterisks (^∗^) highlight significant linear regressions*.

### Seasonal Variations in Carbon Reserves (i.e., Starch Content): Effects of Winter Intensity, Daylight Length, and Latitude

**FIGURE 2:**
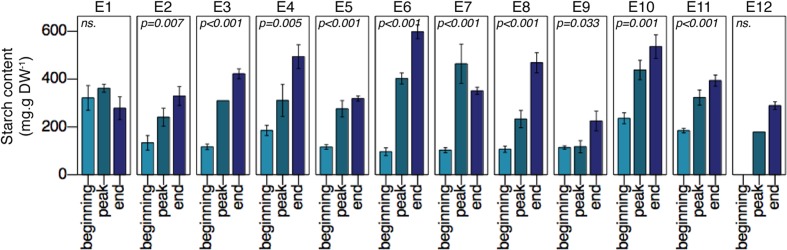
*Zostera noltei* starch content in rhizome over the growing season (three experimental periods on *x*-axis) along a Western European latitudinal gradient (sites E1–E12). Statistical values (*p*-values) indicate significant changes in starch content throughout the experimental period (ns: non-significant effects).

Overall, starch content in *Z. noltei* rhizomes significantly increased over the growing season (**Figure 2**). This was observed at most sites, except at site E1 where no significant changes were detected. At the end of the growing season, starch content in rhizomes reached values between 200 up to 600 mg g DW^-1^ (**Figure 2**), showing a high variability along the latitudinal gradient.

**FIGURE 3:**
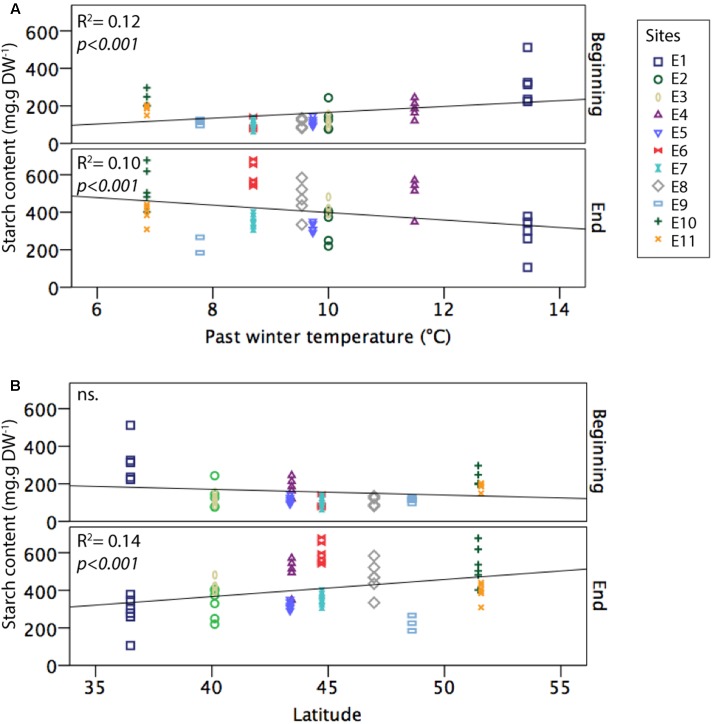
Influence of past winter temperatures **(A)** and latitude **(B)** on *Z. noltei* starch content at the beginning and end of the growing season along the Western European latitudinal gradient (sites E1–E12).

*Zostera noltei* starch content at the beginning of the growing season was weakly but positively related to the averaged past winter temperature (*F* = 13.46, *R*^2^ = 0.1219, *df* = 97, *p* < 0.001) (**Figure 3A**). At the end of the growing season, starch content was weakly but positively related to latitude (*F* = 15.02, *R*^2^ = 0.1403, *df* = 92, *p* < 0.001) (**Figure 3B**). In other words, this suggests that *Z. noltei* populations had greater carbon reserves before winter at the northern sites than at the southern ones. Daylight length positively correlated to starch content at the peak of the growing season (*R*^2^ = 0.137, *F* = 7.152, *p* = 0.001) but did not show any clear effect on starch content at the beginning or at the end of the growing season.

### Effects of Nutrient Enrichment and Small-Scale Disturbance on Carbon Reserves

**FIGURE 4:**
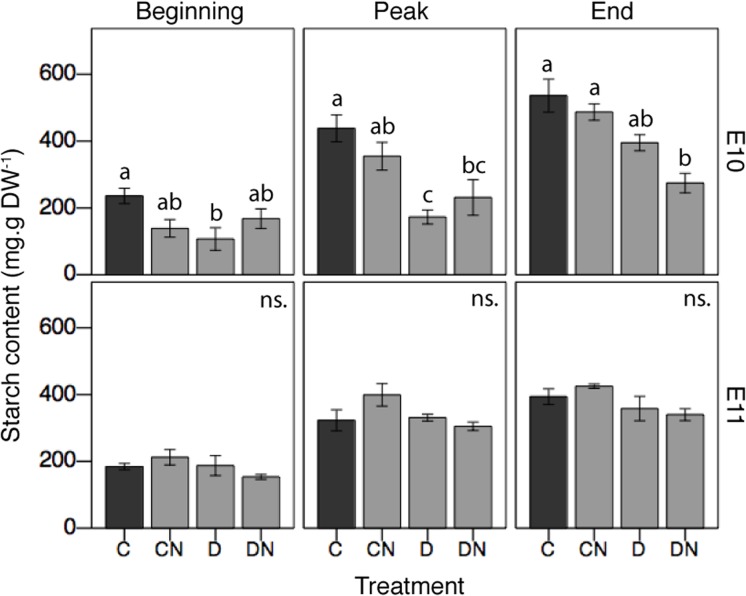
Effects of short-term stress and disturbance events on *Z. noltei* starch content for the different treatments and along the growing season at sites E10 and E11. For the treatments: C = control (i.e., undisturbed-no nutrient); CN = undisturbed with nutrient; D = disturbed-no nutrient; DN = disturbed with nutrient. Small letters (a, b, ab) indicate significant statistical differences among treatments for each site and each experimental period from the *post hoc* Tukey tests.

To investigate the effects of short-term stress events such as nutrient enrichment, small-scale disturbances, such as aboveground biomass removal, and their combination on carbon reserves, two sites located at the same latitude (sites E10 and E11) were subjected to manipulative *in situ* experiments. Treatments and experimental period had different effects at both sites (interaction effects, **Table 3** and **Figure 4**). In controls, starch content was significantly higher at site E10 as compared to site E11 (**Figure 4**). When applied alone, nutrient enrichment had no significant effect on starch content at either sites [Treatment (CN), **Figure 4**], while aboveground biomass removal significantly decreased starch content at site E10 at the beginning and peak of the season [Treatment (D), **Figure 4**]. Aboveground biomass removal alone [Treatment (D)] did not result in a significant change in starch content at site E11 (**Figure 4**). However, when combined, nutrient enrichment and aboveground biomass removal significantly decreased starch content at site E10 at the peak of growth and at the end of the growing season [Treatment (DN), **Figure 4**]. Additionally, the combination of stress and disturbances tended (*p* < 0.1) to reduce starch content at both sites and all times, when compared to control situations [Treatment (DN), **Figure 4**].

**Table 3:** Local effects of short-term stress and small-scale disturbances on *Z. noltei* starch content.

	% TSS	*F*	*p*-value
Experimental period	8	111.807	<0.001^∗^
Treatment	2	19.081	<0.001^∗^
Site	0.008	0.244	0.623
Experimental period ^∗^ Treatment	0.5	2.487	0.029^∗^
Experimental period ^∗^ Site	0.3	4.395	0.015^∗^
Treatment ^∗^ Site	1	9.461	<0.001^∗^
Experimental period ^∗^ Treatment ^∗^ Site	0.4	2.899	0.06

*Sources of variation include three factors: Experimental period (three levels: beginning, peak, and end of the growing season); Treatment (three levels: nutrient enrichment, disturbance, and nutrient enrichment × disturbance); and Site (two levels: E10 and E11). Statistical analysis includes the effects of individual factor and their interactions. The column “%TSS” represents the total sum of squares as percentage (Type III) accounted by each factor. Asterisks (^∗^) highlight factors with significant effects (i.e., p****<****0.05)*.

## Discussion

As dynamic systems, seagrass meadows experience variations in their abiotic and biotic conditions throughout the year. Some are related to seasonal fluctuations and geographical settings; but others are rather local such as short-term nutrient enrichment or small-scale disturbances. In this study, we observed the variation of seagrass carbon reserves (i.e., starch content) in rhizomes throughout the growing season along a latitudinal gradient and how this variation is related to the geographical distribution of seagrasses and seasonal fluctuations. We also had some indications on the effect of short-term local events, suggesting that short-term nutrient enrichment and aboveground biomass removal could reduce the storage of carbon reserves, particularly when combined. Potential implications of these latitudinal and local effects on seagrass long-term survival and resilience will also be discussed in the next sections.

### The Influence of Seasonal Fluctuations

Our study reveals that seasonal fluctuations such as winter intensity or latitude may affect seagrass rhizome carbon reserves, indicating that seagrass carbon reserves at the end of the season are positively related to latitude. In other words, northern seagrass populations accumulate more reserves over their growing season. However, this pattern is opposite at the beginning of the growing season, suggesting that northern seagrass populations have lower carbon reserves at the beginning of the growing season than southern populations. These cross patterns suggest that northern seagrass populations are more dependent on their carbon reserves during winter, but also build-up more reserve during the growing season than southern populations.

In southern Europe, winter temperatures are milder and days lengthier with longer photoperiod and higher daily doses of light (less clouds), allowing a higher photosynthetic production than in northern Europe (Touchette and Burkholder, 2000; Olivé et al., 2007). Those southern seagrass population usually form evergreen meadows whereas, for *Z. noltei* meadows in northern temperate areas in winter, only the below ground biomass, some sparse shoots, and seed banks remain until spring, when new shoots grow again and seedlings emerge (Pérez-Lloréns and Niell, 1993; Auby and Labourg, 1996; Vermaat and Verhagen, 1996; Plus et al., 2003). This likely explains the latitudinal pattern of consumption and larger depletion of carbon reserves (i.e., starch content) observed in the north as compared to the south since, in the north, seagrasses rely on these reserves for maintenance, rather than in photosynthesis to sustain the plant during winter. In a similar way, latitudinal differences and seasonal fluctuations can explain the differences in carbon storage during the growing season because, in summer, temperatures are milder while daily doses of light are longer in northern Europe as compared to southern locations. This hence provides an environment with larger light resources and minor temperature stress for the northern seagrass populations. This is supported partly by our results showing a weak but significant relationship between carbon reserves (i.e., starch content) and daylight length at the peak of the growing season, and by other studies where the combination of mild temperature and high daylight doses (duration of irradiance) has been described as an optimum situation to stimulate a positive growth–respiration balance (Madsen, 1991; Alcoverro et al., 1999; Brun et al., 2003). Additionally, southern seagrass populations are more susceptible of suffering from environmental stress linked to heat waves (Grilo et al., 2011) or excessive daily light irradiance causing photo-inhibition (Jimenez et al., 1987; Schubert et al., 2015). These summer stresses may explain the lack of carbon reserves (i.e., starch content) increase for the most southern site during the growing season. In northern latitudes, carbon reserves are critical for winter survival. However, in southern latitudes, higher winter light availability (daily doses) and milder winter temperatures may allow southern seagrass populations to maintain a positive primary production that favors growth and survival overwinter (Madsen, 1991; Peralta et al., 2005). Winter seagrass growth in southern latitudes also explains the slightly higher carbon reserves (i.e., starch content) at the beginning of the next growing season observed in this study. In summary, seagrass populations in northern latitudes may benefit from the milder summer conditions (high daily light doses, limited stress caused by extreme temperatures or light inhibition) while southern seagrass populations benefit from milder winter conditions, favoring continuous growth, thereby sustaining evergreen populations.

These patterns of higher carbon reserves build-up in northern populations during summer, and stronger depletion during winter, also agree with seagrass reproductive patterns at northern latitudes, where populations tend to be deciduous and invest in higher reproductive effort (Van Tussenbroek et al., 2016). Thus, northern seagrass populations may be considered to be in a perpetual colonizing phase (Peralta et al., 2005). Yearly population survival partly depends on the carbon reserves (i.e., starch content) stored in dormant rhizomes to survive the cold winter conditions, and partly on seed production (Van Tussenbroek et al., 2016). In contrast, southern (evergreen) seagrass population may be considered as dependent on their constant clonal growth and on their carbon reserves only when external resources are limiting the positive balance of their net primary production (Coyer et al., 2004; Zipperle et al., 2011; Ribaudo et al., 2016).

### The Influence of Unpredictable Short-Term Stress Events

Short-term stress and small-scale disturbance events can occur at any time and severely affect seagrass meadows, independently from their geographical distribution. Short-term stress events range from waste water discharge (Cabaço et al., 2007), or algal cover (Han et al., 2016), while small-scale disturbances are related mainly to intense herbivory (Christianen et al., 2014), trampling (Eckrich and Holmquist, 2000), collection of fauna (Cabaço et al., 2005). Our study showed that combined short-term stress and small-scale disturbances, i.e., nutrient enrichment and above-ground removal, may generate a decrease in carbon reserves, i.e., starch content (effect detected at site E10). However, positive effects may sometimes compensate negative effects and environmental differences as seen for instance in Peralta et al. (2006) where a moderate increase in hydrodynamics can stimulate seagrass primary production. This may partly explain the lack of starch response at site E11, more exposed to winds and hydrodynamics (personal observations, Soissons LM) and where carbon reserves could be replenished fast after a disturbance due to an increased productivity (Peralta et al., 2006) and nutrients could be assimilated faster [positive effect of nutrient enrichment; (Brun et al., 2002)].

Despite a significant decrease of their carbon reserve, *Z. noltei* plants from site E10 had higher starch content throughout the growing season than those from site E11. It is therefore also plausible that plants from E10 used their carbon reserve to overcome the combined short-term stress and small-scale disturbance to maintain a vigorous growth. In contrast, the plants from site E11 (experiencing different local conditions) may be able to afford carbon storage with a lower biomass and growth. Working on natural systems implies to cope with additional effects associated with natural variability that are difficult to identify, but also provides a much more realistic frame for treatment responses. Divergences in the plants’ response between sites E10 and E11 reflect differences in environmental conditions and/or among populations. However, our results still support that the combination of short-term stress and disturbance may potentially deplete seagrass carbon reserves.

Our results agree with responses found for other temperate seagrass species such as *Posidonia oceanica*, which carbon reserves decreased after stress events (Genot et al., 1994; Ruiz and Romero, 2003). Previous studies stated that soluble carbohydrate concentrations such as starch may not only be a valid indicator for seagrass growing success after long-time stress periods (Govers et al., 2015), but also for seagrass resilience (i.e., response to short-term stresses and disturbances). Carbon reserve pools and clonal growth rates are recognized as key elements for seagrass recovery (Hemminga and Duarte, 2000; Borum et al., 2004). However, both may vary widely among seagrass species (Ralph et al., 2007). Therefore, the amount of starch needed to overcome a short-term stresses and/or small-scale disturbance must also be species dependent.

### Potential Implications for Seagrass Meadows in a Changing Environment

Integrating the results of the latitudinal gradient with those of the stress-disturbance experiments suggest that northern seagrass populations might be more sensitive to short-term stress and disturbance events, as seagrass cope with this type of events by using their carbon reserves, which are fundamental to withstand colder winters at high latitudes. Present findings have implications for seagrass meadows under changing conditions, where unpredictable (stochastic) events due to climate change (Short and Neckles, 1999; Easterling et al., 2000; Thomson et al., 2015) or the increase of anthropogenic stressors on coastal ecosystems (Halpern et al., 2008; Duarte, 2014) are expected to become more frequent and of higher intensity. Indeed, although seagrasses have the capacity to acclimate to seasonal and latitudinal fluctuations (Peralta et al., 2005; Cabaço et al., 2009; Staehr and Borum, 2011; this study), our study shows that the combination of short-term stress and disturbance events may reduce seagrass carbon reserves, needed in general for overwintering. This may be particularly important as high carbon reserves are central to overwintering at northern latitudes (Govers et al., 2015).

## Author Contributions

LS, MvK, PH, and TB conceived and designed the experiment. LS, EH, RA, IA, LB, FB, PC, ND, JF, FG, J-MG, LG, TG, PK, BO, GP, AP, MR, LR, and MV execution and analysis of European sampling and manipulative experiment. LS, MvK, and TB wrote the manuscript, other authors provided editorial advise.

## Conflict of Interest Statement

The authors declare that the research was conducted in the absence of any commercial or financial relationships that could be construed as a potential conflict of interest.
